# The role of local versus biogeographical processes in influencing diversity and body‐size variation in mammal assemblages

**DOI:** 10.1002/ece3.1978

**Published:** 2016-02-03

**Authors:** Luiz Carlos S. Lopez, Marcos S. L. Figueiredo, Maria Paula de Aguiar Fracasso, Daniel Oliveira Mesquita, Ulisses Umbelino Anjos, Carlos Eduardo Viveiros Grelle

**Affiliations:** ^1^ Departamento de Sistemática e EcologiaCentro de Ciências Exatas e da NaturezaUFPBJoao PessoaBrazil; ^2^ Departamento de EcologiaCentro de Ciências da SaúdeUFRJRio de JaneiroBrazil; ^3^ Departamento de EstatísticaCentro de Ciências Exatas e da NaturezaUFPBJoao PessoaBrazil

**Keywords:** body size, fractal niche, mammals, skewness, slope, vicariance

## Abstract

Our objective was to estimate and analyze the body‐size distribution parameters of terrestrial mammal assemblages at different spatial scales, and to determine whether these parameters are controlled by local ecological processes or by larger‐scale ones. Based on 93 local assemblages, plus the complete mammal assemblage from three continents (Africa, North, and South America), we estimated three key distribution parameters (diversity/size slope, skewness, and modal size) and compared the values to those expected if size distributions are mainly controlled by local interactions. Mammal diversity decreased much faster as body size increased than predicted by fractal niche theory, both at continental and at local scales, with continental distributions showing steeper slopes than the localities within them. South America showed a steeper slope (after controlling for species diversity), compared to Africa and North America, at local and continental scales. We also found that skewness and modal body size can show strikingly different correlations with predictor variables, such as species richness and median size, depending on the use of untransformed versus log‐transformed data, due to changes in the distribution density generated by log‐transformation. The main differences in slope, skewness, and modal size between local and continental scales appear to arise from the same biogeographical process, where small‐sized species increase in diversity much faster (due to higher spatial turnover rates) than large‐sized species. This process, which can operate even in the absence of competitive saturation at local scales, generates continental assemblages with steeper slopes, smaller modal sizes, and higher right skewness (toward small‐sized species) compared to local communities. In addition, historical factors can also affect the size distribution slopes, which are significantly steeper, in South American mammal assemblages (probably due to stronger megafauna extinction events in South America) than those in North America and Africa.

## Introduction

The patterns found in size‐diversity distributions have intrigued researchers for decades and have resulted in several hypothesis that attempt to explain the processes that control the variation in species diversity along the size spectrum (Hutchinson and Macarthur 1959; Brown 1995; Allen et al. 2006). Some of these hypotheses, unfortunately, have not been subjected to systematic testing or have been tested using methodologies that are known today to present some bias and inaccuracy problems. For example, May (1978) proposed that the increasing (“fractal”) niche partitioning as organism size decreases is one of the main forces that controls the variation in biodiversity along the size continuum. According to May's predictions, species diversity should decrease following an allometric slope of −2/3 in response to an increase in body mass (or −2 if body length is used instead of body weight). This prediction, unfortunately, has not until now, been systematically tested (using several assemblages in different parts of the world) for mammals and previous attempts to calculate the slope of size–diversity distributions have used histograms composed of simple logarithm binned data, a method that previous studies (with other similar frequency distributions) have shown to produce inaccurate slope estimations (Loder et al. 1997; Bonnet et al. 2001; Edwards 2008; White et al. 2008; Clauset et al. 2009a).

An alternative hypothesis to explain the size–diversity distributions is diffusional models such as the ones proposed by Clauset et al. (2009b) and Clauset (2013), where the evolutionary shift of organisms toward larger sizes (“Cope's rule”) coupled with an increased probability of extinction faced by large‐sized species is the main processes that control size–diversity distributions. Clauset et al. (2009b) model appears to successfully fit empirical data obtained from mammal and bird databases (Clauset et al. 2009b; Clauset 2013). However, the diffusional model, similar to the fractal niche one, does not offer predictions about how the spatial scale (e.g., local vs. regional) affects the rate of diversity decay in response to body‐size increase.

Another key parameter to understand the forces that structure diversity‐size distribution is the skewness (the degree of distribution symmetry). Body‐size distributions obtained from large spatial scales (regional or continental biotas) show in general, positive values of skewness, which indicates nonsymmetric communities skewed toward small‐sized species (“right skewed”). Local assemblages, in contrast, can show a skewness that is close to zero (uniform symmetric distribution) or even has a negative value of skewness (“left skewed,” representing nonsymmetric communities dominated by large‐sized species) (Kozlowski and Gawelczyk 2002). This pattern of skewness variation at different spatial scales has been explained, until now, as the result of local competitive forces and niche partitioning (as for the fractal niche theory), where size competitive exclusion makes local assemblages more uniformly distributed compared to those on regional spatial scales (Brown and Nicoletto 1991; Gaston and Blackburn 2000; Kozlowski and Gawelczyk 2002; Allen et al. 2006). However, we propose that differences in body‐size skewness between different spatial scales do not necessarily imply competitive filter acting at local scales, because evolutionary large‐scale processes, such as higher vicariance rates associated with small body sizes, can also lead to difference in skewness between local and regional scales.

It is also not clear to what extent body‐size skewness is correlated with the diversity and latitude of the community studied, which is a crucial question to understand how large‐scale processes [such as the “Bergmann's rule”(Bergmann 1848)] influence size distribution symmetry. Some studies have identified strong correlations between body‐size skewness, species diversity, and/or latitude (Knouft 2004; Olson et al. 2009), whereas other studies have identified a weaker nonsignificant correlation between the same parameters (Ulrich and Fiera 2010). However, comparisons among studies concerning body‐size skewness are difficult, because some of them use raw data to calculate the skewness (“linear skewness”), whereas others log‐transform the data prior to calculation (“log skewness”). This difference in data treatment can confound the comparison among results because log‐transformation can change the body‐size distribution symmetry and shift it toward large sizes (Thibault et al. 2011).

Another hypothesis regarding diversity and body size that lacks adequate testing is the similarity of modal body size among continental mammal assemblages (Brown et al. 1993). Brown et al. (1993) proposed that energetic trade‐offs tend to converge body sizes toward the same modal diversity optimum (the “optimum body size” for mammals about 100 g) at continental scales, whereas competitive exclusion would change the local distribution mode away from 100 g (as competition does to local body‐size skewness). Previous attempts to verify whether mammal assemblages in different continents converge to the same modal size, around 100 g, used visual inspection of log‐binned histograms, which can provide inexact estimations of modal sizes compared to analytical methods (Bickel and Fruhwirth 2006). Hypotheses that generate predictions regarding modal sizes are also complicated because, similar to skewness, the same size distribution can show different modal sizes if data are log‐transformed or not (and depending on which log base was used) prior to analysis, can lead to different interpretations depending on the use of linear or log‐transformed data in the analysis (Loder et al. 1997; Gaston and Blackburn 2000; Thibault et al. 2011).

In this study, we present a systematic comparison among these key size distribution parameters (slope, skewness, and modal size) obtained from terrestrial mammal assemblages from 93 localities on three different continents to estimate how these parameters vary between different spatial scales (local vs. continental) and among continents. Our aim was to determine whether the variation in these distribution parameters supports the hypothesis of fractal niche partitioning (May 1986) and energetic trade‐offs (Brown et al. 1993) as the main forces governing mammal body‐size distribution.

As an alternative, we explore the possibility that the slope, skew, and mode of mammal body‐size distributions are strong interlinked parameters that can be simultaneously affected by processes that change the proportion of large size species at regional scales. For example, according to Brown & Maurer (1989), terrestrial mammals, in general, display a positive correlation between body and species geographic range. If large‐sized species tend to occupy larger geographical areas, their diversity will increase less than the diversity of smaller‐sized ones when one move from local to regional scales, because expanding the database spatial scale will encompass more new small‐sized species ranges than new large‐sized ones, leading to regional assemblages with reduced proportions of large size species (Brown and Nicoletto 1991).

We also performed the first comparative study using both the linear and log‐transformed skewness and modal size estimates of mammal assemblages. Although the basic mathematical effects of log‐transformation were well known, the way these transformations affect the interactions among ecological variables related to body size are poorly understood. As a result, we tried to determine which kind of data format (linear vs. log‐transformed) can capture better the interaction among different parameters.

## Materials and Methods

### Body‐size database

**Figure 1:**
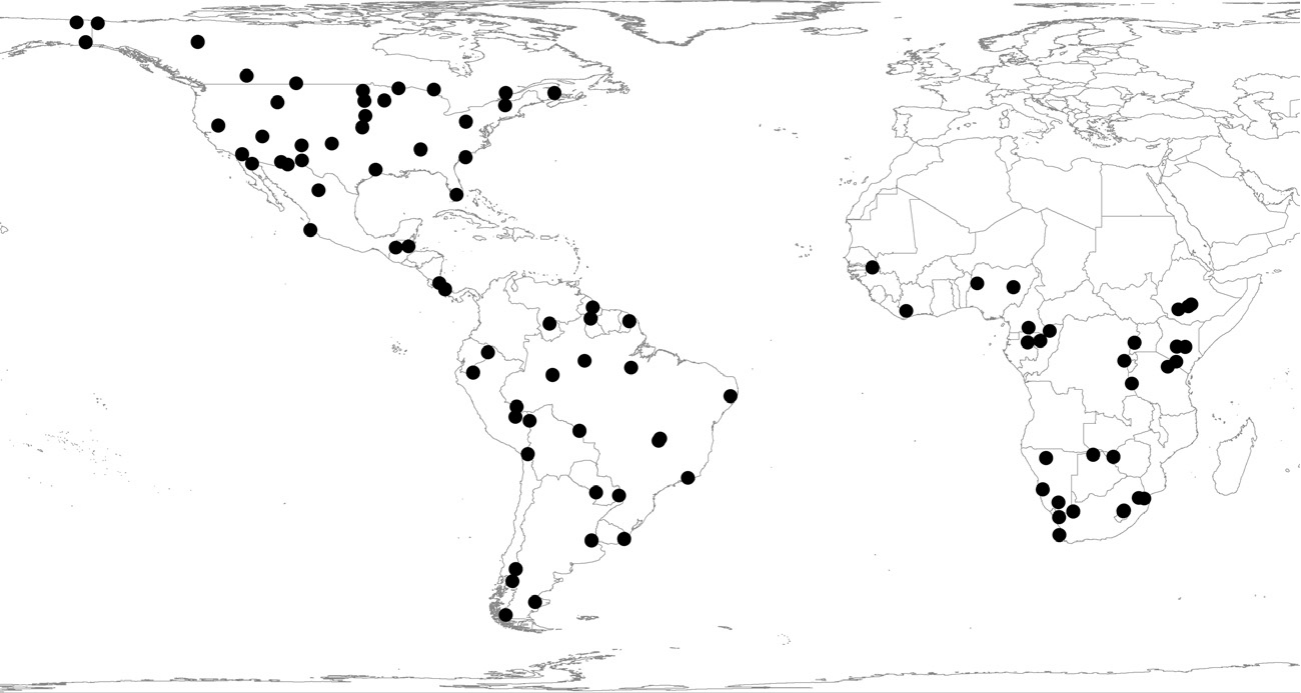
Localities used in the analysis (*n* = 93). Body‐size distribution parameters were estimated for localities belong to three large biogeographical regions: Afrotropical (“Africa,” *n* = 30), Nearctic (“North America,” *n* = 35), and Neotropical (“South America,” *n* = 28). Detailed information about each site can be found at Appendix S1.

To make our results comparable with those of previous studies, our analysis was restricted to terrestrial nonvolant mammals. We estimated the size distribution parameters for 93 localities divided in 3 large biogeographical regions: Afrotropical (“Africa,” *n* = 30), Nearctic (“North America,”) *n* = 35), and Neotropical (‘South America,” *n* = 28) (more detailed information for each locality can be found in Appendix S1) (Fig. 1).

The continental African database was obtained from Kelt and Meyer (2009), and the North and South American mammal body sizes were provided by Rodriguez et al. (2008). Species lists for localities on these three continents were obtained from Brown and Nicoletto (1991), Bakker and Kelt (2000). Kelt and Meyer (2009) and from the Biological Inventories of the World Protected Areas (BIWPA, http://www.ice.ucdavis.edu/bioinventory/bioinventory.html) (Appendix S1).

### Parameter estimation

In order to allow comparisons between our results and the expected value, according to the fractal niche hypothesis, slope was estimated using the original method proposed by May (1978) with corrections to remove the biases caused by rounding procedures and the effects of increasing log interval width. Regressions were performed between the number of species found in log_2_ size class intervals and the middle point of size each interval. For each size distribution, the slope was calculated using three different interval rounding procedures (upper unit, lower unit, and 0.5 unit rounding) and averaged to avoid rounding (Bobe and Behrensmeyer 2004; Kelt and Meyer 2009). Previous work showed that distribution analyses that use the number of events found inside of log‐transformed intervals (like the number of species found inside a given bin size interval) are biased because larger intervals tend to contain more events (e. g. species) just by chance (Bonnet et al. 2001; Edwards 2008; White et al. 2008). In order to correct for this chance effect, the number of species found on each interval was divided by the bin interval width (Bonnet et al. 2001).

The skewness of localities and continents was estimated using both linear and log_2_‐transformed data, according to Zar (1999). Modal sizes for localities and continents were analytically estimated using the method proposed by Bickel and Fruhwirth (2006) implemented in the R package “modest,” to reduce the imprecision caused by the traditional method of simple log‐bins histograms. Similar to the skewness, modal size was calculated using both untransformed data and log_2_‐transformed data. Differences among continent median values of slope, skewness, and modal size were tested with Kruskal–Wallis followed by a median post hoc comparison.

To understand which factors correlate with these three parameters, we performed a semi‐partial Spearman correlation (since some parameters are not normal) between the slope, skewness, and modal of size and three predictor factors: the continent where the locality belongs (categorical factor), the species richness, and the median species body size of each locality (*n* = 93). The partial Spearman correlation of each predictor was estimated using the method implemented by Kim and Yi (2007) in the R package “ppcor.”

## Results

### Slopes

**Figure 2:**
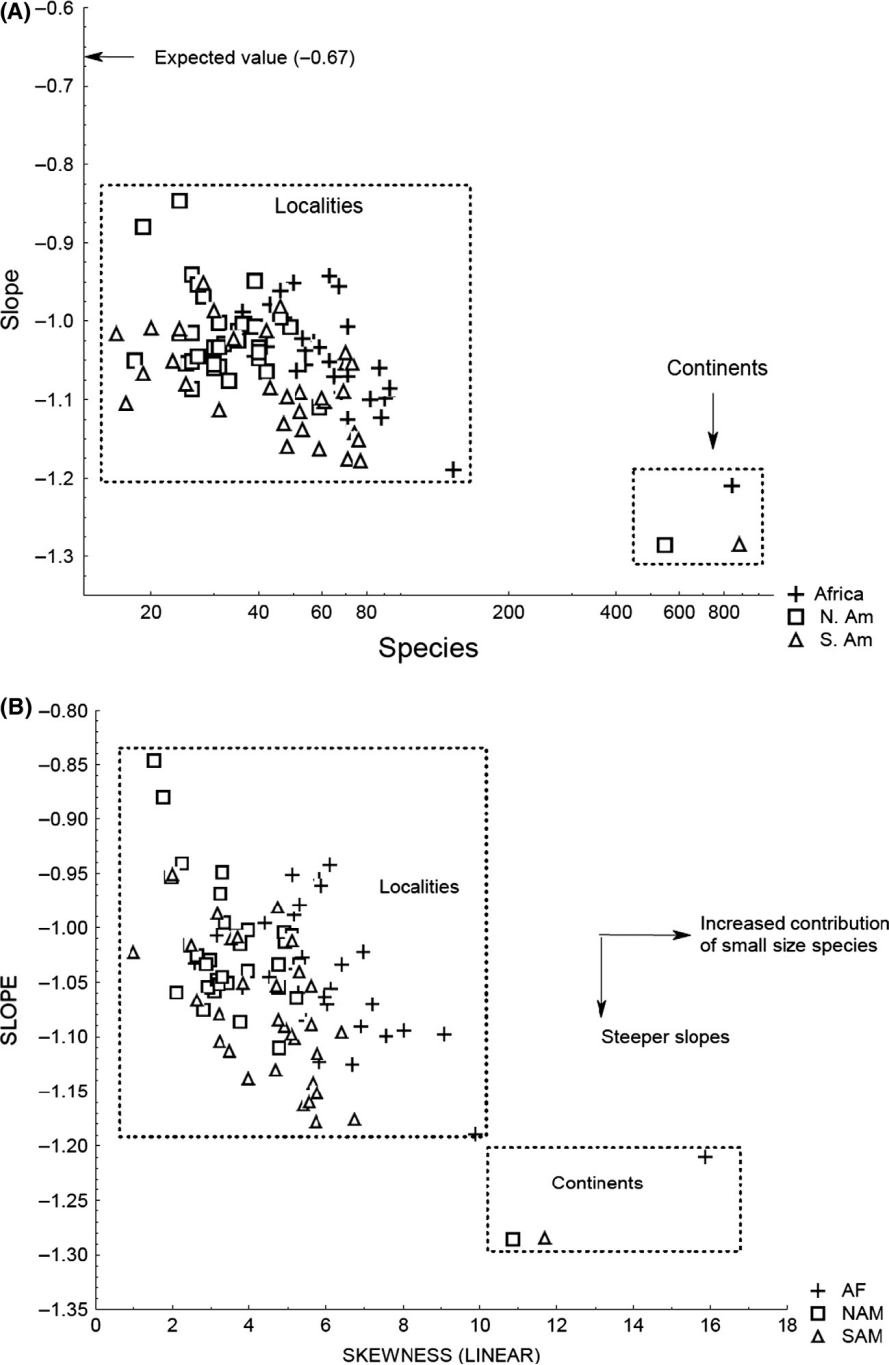
Scatterplot between the rate of diversity reduction as size increases (slope), the number of species (A) and the skewness (B). Both localities and continents loose diversity much faster, as size increase, than the slope predicted by the fractal niche hypothesis (−0.67) (A). Continents present an even more negative slope, compared to localities, because they have a higher proportion of small size species [higher skewness (B)], leading to a faster reduction of diversity as size increase. Notice that South American localities (triangles) display more negative slopes compared to localities in Africa and North America with similar number of species and skewness. The correlation between slope versus species richness(*r* = −0.56, *P* < 0.0001, *n* = 96) and slope versus skewness (*r* = −0.62, *P* < 0.0001, *n* = 96) was both highly significant.

**Figure 3:**
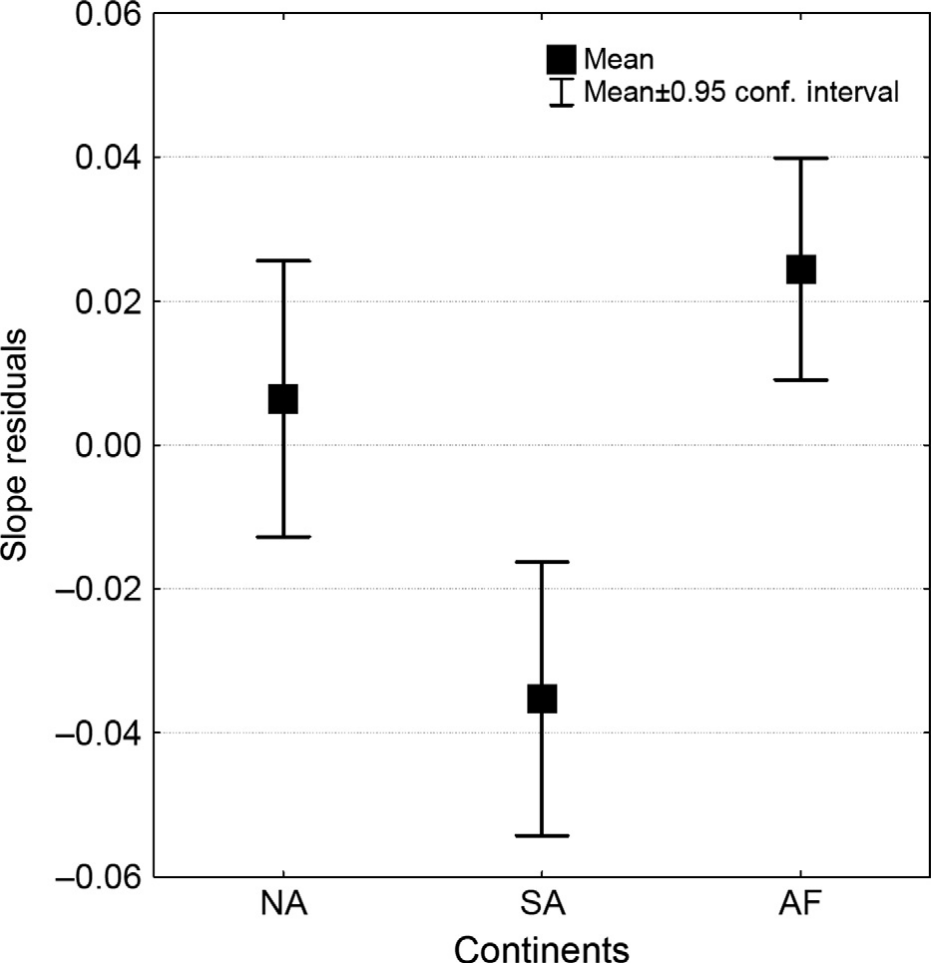
Diversity/size slope residuals (controlled for the variation in species numbers) among 93 localities in three continents. South American localities (SA) presented more negative residual slopes compared to Africa (AF) and North America (NA), indicating that in South America, the diversity of mammals tends to decrease faster when body size increases. South America localities differ from North America and Africa by lacking both very small mammals (insectivores are poorly represented in the continent) and very large ones leading to a “compressed” body‐size distribution with steeper a slope.

The size/diversity slopes, estimated for the localities and the continents, were all more steep (more negative) and significantly different compared to the value proposed by May (1978) (*t*‐test *P *<* *0.001) (Table 1). In addition, the slopes from continental scales were significantly steeper than those estimated from localities (Table 1, Fig. 2). Slope from localities presented highly significant partial correlations with the median size, the species richness, and the continent factor. After control for species richness, localities in South America showed slopes that were steeper compared to those of North American and African localities (Kruskal Wallis KW‐H_(2,93)_ = 18.75 *P* = 0.00008) (Figs. 2, 3).

**Table 1:** Mean (± SD) parameter values for body‐size distributions obtained from terrestrial mammal assemblages located on three continents (Africa, North, and South America) compared with 93 localities within these continents

Distribution parameter	Continental mean (± SD)	Localities mean (± SD)
Slope	−1.26 (± 0.04)	−1.05 (± 0.06) (≠ −0.67 ***)
Skewness (Linear)	12.81 (± 2.68)	4.56 (± 1.66) (**)
Skewness (Log_2_)	0.85 (± 0.08)	0.05 (± 0.29) (**)
Modal size (Linear)	16 g (± 10)	46 g (± 92) (ns)
Modal size (Log_2_)	54 g (± 20)	5993 g (± 10,050) (ns)
Median size	104 g (± 46)	2358 g (± 3042) (**)
% species <100 g	51% (± 5)	28% (± 10) (**)

Asterisks represent a significant difference between continental and local‐scale parameters (**) *P *<* *0.01; (***) *P *<* *0.001)

### Skewness

**Figure 4:**
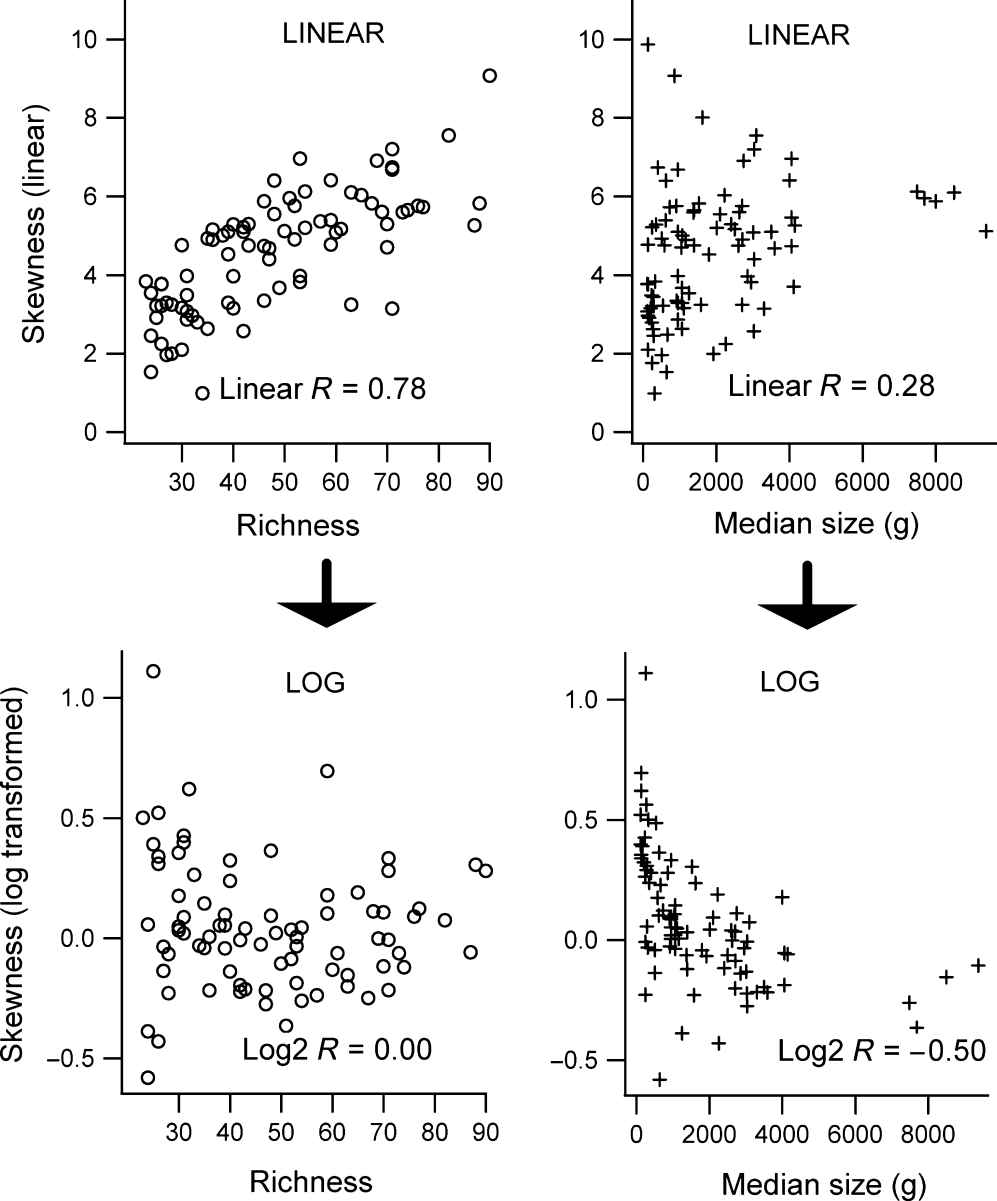
Scatterplots relating the skewness (g1) of terrestrial mammal assemblages in 93 localities in Africa and the Americas with species richness (circles) and median size at each locality (crosses) (positive values of skewness represent assemblages skewed toward small‐sized mammals). In the two upper graphs, skewness was calculated using untransformed (linear) data, while in the lower graphs, the same data were log_2_‐transformed before skewness calculation. Linear skewness shows a strong correlation with the number of species present in each locality, with localities with more species being more skewed toward small sizes (positive “right” skew), but having a weak positive correlation with median size. The same data after log‐transformation show the inverse pattern: a strong negative correlation with median size and a weak (zero correlation) response to locality richness. This striking difference between linear and log skewness arises because log‐transformation tends to “normalize” the distributions, shifting the log skewness toward larger sizes (see Table 2 for partial multiple correlation partial contributions).

Skewness calculated using both the untransformed data (linear skewness) and the skewness obtained from the same data after log_2_‐transformation (log_2_ skewness) differed between local and continental scales (Table 1), with continental mammal assemblages being significantly more skewed toward smaller‐sized species (“right skewed” more positive values of g1 skewness) (Table 1). However, log_2_ data were much less skewed than linear data and some localities showed a negative skewness (left skewed; Table 1, Fig. 4). The correlation between linear skewness and log_2_ skewness estimated for the same data obtained from 93 studied localities was very weak and nonsignificant (*r *=* *0.04, *P *=* *0.70).

The species richness presented a strong, and highly significant, correlation with linear skewness among localities, whereas the continental factor and median size were less important and nonsignificant (Table 2). African localities showed the highest linear skewness, followed by South American and North American localities. The difference in linear skewness among continents was significant (Tables 2 and 3; Fig. 4).

**Table 2:** Spearman semi‐partial correlations between the continent, median size, and species richness and different distribution parameters estimated from 93 mammal assemblages located in Africa, North, and South America

Distribution parameter		Regressor (Spearman partial correlation)
Slope	~	Median (0.53)*** − Richness (0.53)*** + Continents (0.43)***
Skewness (Linear)	~	Richness (0.50)*** + Continents (0.11) + Median (0.03)
Skewness (Log_2_)	~	(−) Median (0.58)*** + Continents (0.20)* + Richness (0.15)
Modal Size (Linear)	~	Continents (0.44)*** + Median (0.32)** − Richness (0.04)
Modal Size (Log_2_)	~	Median (0.48)*** − Richness (0.04) + Continents (0.02)

(*) *P *<* *0.05; (**) *P *<* *0.01; (***) *P *<* *0.001)

**Table 3:** Comparison of body‐size distribution parameters among 93 local mammal assemblages (terrestrial) from Africa (AFR), North America (NAM), and South America (SAM)

Distribution parameter	Continental differences
Slope	NAM^a^>AFR^ab^>SAM^b^(***)
Skewness (linear)	AFR^a^>SAM^b^ >NAM^c^(***)
Skewness (log_2_)	NAM^a^>SAM^ab^ >AFR^b^(*)
Modal Size (linear)	SAM^a^>NAM^b^ >AFR^b^(***)
Modal Size (log_2_)	AFR^a^>SAM^b^ >NAM^b^(***)

Different lowercase letters represent significant differences (*P *<* *0.05) among continents (post hoc Median test). Asterisks represent the overall significance level of Kruskal–Wallis.

In contrast to the linear skewness, the log_2_ skewness responded strongly and significantly to variations in mammal median size, followed by the effects of the continental factors, richness (Table 2). North American localities showed the highest log_2_ skewness, followed by South American and African localities, and the difference among continents was significant (Tables 2 and 3, Fig. 4).

### Modal mammal size

**Figure 5:**
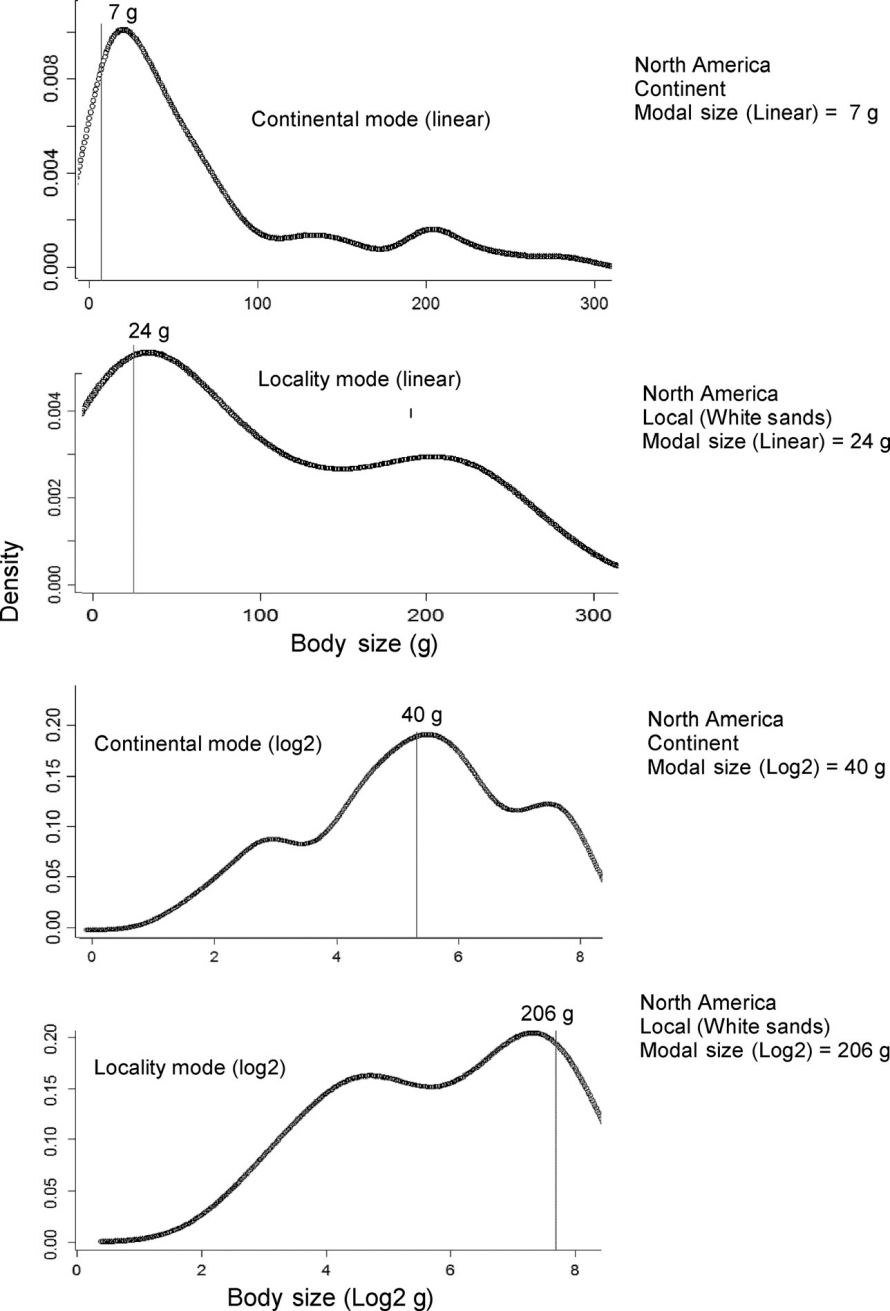
Modal mammal size of the North American Continent compared to one locality within the same continent (White Sands). The modal sizes were analytically estimated using untransformed size data (linear) and the same data after log_2_‐transformation. Local mammal diversity peaks at larger body sizes compared to the continent as a whole. This same pattern is found in other localities from the Americas and Africa (Table 1) and is probably the result of a faster accumulation of small‐sized species at larger spatial scales. Log_2_‐transformed data show higher model values compared to the same data prior to transformation because log‐transformation shifts the distribution toward large sizes (the figure displays the R package “modest” graphic output kernels between 1 and 256 g (2^8^) to facilitate the visual comparison between linear and log_2_ modal sizes).

Continental modal sizes were smaller than local modes using both linear and log‐transformed data, but the difference between local and continental modes was much stronger using log_2_ data (54 g for local vs. 5334 g for continental distributions). Both log_2_ (54 g) and linear mean continental modes (16 g) were well below the expected value of 100 g proposed by Brown's theory of optimal energetic size for mammals (Table 1, Fig. 5).

The locality modal sizes (*n* = 93) displayed a weak and nonsignificant correlation between the modes estimated using linear data and the same data after log_2_‐transformation (*r *=* *0.13, *P *=* *0.21). Partial correlations analysis found that locality modes estimated using linear data were significantly correlated to the continental factor and the median size of the distribution, while the correlation with richness presented a weaker, nonsignificant, effect (Table 2). In contrast, the mode obtained from log‐transformed data presented a highly significant correlation only with median size. Continents also are ranked in a different way when linear data were used to calculate the modal size compared to the rank order obtained using the log‐transformed modal values, with South America localities presenting the highest linear modal sizes, while African localities presented the highest log modes (Table 3).

## Discussion

Our analyses found that species diversity increased much faster as mammal body size decreased, than expected by the fractal niche‐partitioning hypothesis (May 1978, 1986), indicating that other factors besides fractal niche‐partitioning affect the slope of mammalian body distributions. The difference between the observed and the expected slope, according to the fractal model, can lead to a dramatic increase in the predicted biodiversity of small‐sized organisms. For example, the expected slope of −2/3 (−0.67) implies that each halving in body weight leads to an increase of 59% in the number of species, whereas the observed slope for continental mammal assemblages (−1.26) implies in an increase of 139% in species diversity for each halving in body size.

We also identified that the increase in the rate of diversity due to size reduction is affected by the spatial scale used, as first noticed by Brown and Nicoletto (1991) and by biogeographical differences among continents (Tables 1 and 2). The slopes of the continental mammal assemblages are significantly more negative compared to those of local assemblages within the same continents (Table 1, Fig. 2), indicating that diversity increases faster in response to size reduction at larger spatial scales. As a result, continents show a higher proportion of small mammal species than local assemblages located within them (Table 1, Fig. 2). This pattern might be the result of large size species presenting larger geographical distributions compared to small size ones, leading to a faster geographical turnover of small size species (Brown 1995).

After controlling for differences in richness, South American localities acquire diversity significantly much faster as body size decreases, compared to those in Africa and North America (Fig. 2), suggesting that South American mammal assemblages display a distinctive, probably historical, slope that relates body size and diversity (Marquet and Cofre 1999; Morales‐Castilla et al. 2012). The South American mammal biota strongly differs from that of Africa and North America in terms of body size, due to the absence of both very small (the small insectivores have poorly dispersed in South America after the formation of Panama Isthmus) and very large mammals (because the South American megafauna suffered from stronger events of pleistocenic and pliocenic extinction), leading to a “compressed” size distribution where diversity decreases with a steeper slope along the shorter span of size variation (Fig. 2), (Smith et al. 2004; Smith and Lyons 2011).

Regarding the skewness (the degree of distribution symmetry of mammal body sizes), our findings confirm the well‐known pattern where continental distributions are more skewed (more positive g1) toward small‐sized species than toward local distributions (Table 1). This pattern has intrigued researchers for decades and usually has been explained as a result of local processes such as competitive exclusion among species of a similar size (Kozlowski and Gawelczyk 2002; Allen et al. 2006). We propose that the pattern can be explained by the same process we used to explain the differences among slopes at different scales: a larger proportion of small‐sized species at larger spatial scales, due to faster vicariance speciation. A faster spatial turnover of small‐sized species can explain why continental assemblages are more skewed toward small species, without assuming that local sites are necessarily saturated with species (a prerequisite for explanations based on competitive exclusion).

Understanding the processes that control the variation in skewness among localities in different continents is more complex, because we found that the way the skewness is calculated (using linear or log‐transformed data) will deeply affect the way that skewness varies among sites. We found that linear skewness is strongly positively correlated with the mammalian species richness found at each locality (this result supports similar findings of Olson et al. (2009), using bird size distributions inferred from cell grids around the globe). As a result, African localities tend to show higher values of linear skewness, probably because they contain more species, on average, than North and South American sites (Table 3).

The skewness obtained from log‐transformed data, in contrast to linear skewness, is strongly and negatively correlated with median size, with a weaker, nonsignificant, influence of species richness (Table 2, Fig. 3). This occurs because log‐transformation tends to shift the distribution toward larger size classes (log‐transformation is a common tool to transform a right‐skewed distribution into a normal distribution), making log‐transformed data more sensitive to the presence of larger species (which is associated with larger median sizes). This negative correlation between log skewness and median size probably explains why North American sites show higher log skewness values, because they also have lower median sizes (Table 3).

The variation in modal sizes at different spatial scales also supports our hypothesis that a differential spatial turnover related to body size is one of the main forces that drives the shape of body‐size distributions at large spatial scales, because each continent has a modal size smaller than the mean modal size found for the localities within them (Table 1). This pattern is expected if continental assemblages contain a higher proportion of small‐sized species compared to localities.

Higher geographical turnover rates associated with smaller body sizes can arise because small organisms tend to present higher population densities, allowing them to generate new viable populations after they become isolated in the beginning of allopatric speciation events. Large size organisms, by contrast, are more prone to suffer local extinctions (due to low population densities) when their populations are geographically isolated, resulting in a lower rate of speciation by vicariance. The effects of body size on generation time and dispersal rates also can enhance vicariance rates of small size organisms. Larger size organisms also tend to disperse more than small size ones (Peters 1983; Brown and Nicoletto 1991; Gaston and Blackburn 2000).

Similar to skewness, the modal size obtained using linear data is very different from that obtained from log‐transformed data (Table 1, Fig. 4), with the log_2_ modes showing higher values than linear ones (especially at local scales). The difference between the linear and log modal size probably reflects the effects of log‐transformation, which shifts the distributions toward large sizes and makes the log mode more sensitive to the presence of large‐sized species.

Localities in South America showed the highest linear modal sizes compared to those in Africa and North America, probably due to the low diversity of small insectivores in the Neotropics (Smith and Lyons 2011). Log‐transformed modal sizes, in contrast, are higher in African localities, which have a larger median size, due to the African megafauna. This provides evidence that modal size, similar to skewness, is strongly affected by the use of linear versus log‐transformed data, with a linear mode responding to changes in the modal size of smaller species, whereas modes estimated using log‐transformed data are shifted toward, and are strongly affected by, the presence of large‐sized species. Despite these differences, both linear‐ and log‐continental modal size values were well below the expected value of 100 g according to the Brown hypotheses of optimal energetic size, indicating the presence of other forces controlling the continental modal size in mammals (Raia et al. 2010).

In conclusion, we propose that mammal size distributions acquire much more diversity as size decreases than would be expected if the fractal niche‐partitioning scenario is the main force controlling the relationship between body size and diversity, especially at large spatial scales. Consequently other forces, like the evolutionary “diffusion” of species (followed by the increase in the extinction rate) toward large sizes (Clauset et al. 2009b), probably are important to determine the diversity‐size slope, although this alternative hypothesis was not explicitly tested here and deserves future analysis.

We also propose that the main differences between continental and local body‐size distributions in terms of slope, skewness, and modal size can be explained by the same main process: continental distributions contain a higher proportion of small size species compared to local ones, indicating the presence of a faster spatial turnover of small‐sized taxa. Consequently, understanding the processes linking variations in species spatial turnover with body size is crucial to understanding why body‐size distributions change along different spatial scales.

We also demonstrated that large‐scale factors (probably historical) can significantly affect the slope and other parameters related to body‐size distributions, as well as the use of linear or log‐transformed data. Although the normalization effect of log‐transformation is well‐known data analysis, we found that log‐transformation can also have strong unpredictable effects on the sensitivity of skewness and modal size to different aspects of body‐size distributions. We suggest that such effects should be considered in the future research about diversity‐size distributions.

## Conflict of Interest

None declared.

## Supporting information


**Appendix S1** List of localities used in the analysis.

## References

[ece31978-bib-0002] Allen, C. R. , A. S. Garmestani , T. D. Havlicek , P. A. Marquet , G. D. Peterson , C. Restrepo , et al. 2006 Patterns in body mass distributions: sifting among alternative hypotheses. Ecol. Lett. 9:630–643. 16643307 10.1111/j.1461-0248.2006.00902.x

[ece31978-bib-0003] Bakker, V. J. , and D. A. Kelt . 2000 Scale‐dependent patterns in body size distributions of neotropical mammals. Ecology 81:3530–3547.

[ece31978-bib-0004] Bergmann, C. 1848 Über die Verhältnisse der Wärmeökonomie der Thiere zu ihrer Grösse. Abgedruckt aus den Göttinger Studien 1847. – Vandenhoeck and Ruprecht.

[ece31978-bib-0005] Bickel, D. R. , and R. Fruhwirth . 2006 On a fast, robust estimator of the mode: comparisons to other robust estimators with applications. Comput. Stat. Data Anal. 50:3500–3530.

[ece31978-bib-0006] Bobe, R. , and A. K. Behrensmeyer . 2004 The expansion of grassland ecosystems in Africa in relation to mammalian evolution and the origin of the genus Homo. Palaeogeogr. Palaeoclimatol. Palaeoecol. 207:399–420.

[ece31978-bib-0007] Bonnet, E. , O. Bour , N. E. Odling , P. Davy , I. Main , P. Cowie , et al. 2001 Scaling of fracture systems in geological media. Rev. Geophys. 39:347–383.

[ece31978-bib-0101] Brown, J. H. , and B. A. Maurer . 1989 Macroecology: the division of food and space among species on continents. Science 243:1145–1150. 17799895 10.1126/science.243.4895.1145

[ece31978-bib-0008] Brown, J. H. 1995 Macroecology. University of Chicago Press, Chicago.

[ece31978-bib-0009] Brown, J. H. , and P. F. Nicoletto . 1991 Spatial scaling of species composition – body masses of north‐american land mammals. Am. Nat. 138:1478–1512.

[ece31978-bib-0010] Brown, J. H. , P. A. Marquet , and M. L. Taper . 1993 Evolution of body‐size – consequences of an energetic definition of fitness. Am. Nat. 142:573–584. 19425961 10.1086/285558

[ece31978-bib-0011] Clauset, A. 2013 How large should whales be? PLoS ONE 8:e53967. 23342050 10.1371/journal.pone.0053967 PMC3546790

[ece31978-bib-0012] Clauset, A. , C. R. Shalizi , and M. E. J. Newman . 2009a Power‐law distributions in empirical data. Siam Rev. 51:661–703.

[ece31978-bib-0013] Clauset, A. , D. J. Schwab , and S. Redner . 2009b How many species have mass M? Am. Nat. 173:256–263. 19090772 10.1086/595760

[ece31978-bib-0014] Edwards, A. M. 2008 Using likelihood to test for Levy flight search patterns and for general power‐law distributions in nature. J. Anim. Ecol. 77:1212–1222. 18631370 10.1111/j.1365-2656.2008.01428.x

[ece31978-bib-0015] Gaston, K. J. , and T. M. Blackburn . 2000 Pattern and Process in Macroecology. Blackwell Science, Oxford, UK.

[ece31978-bib-0016] Hutchinson, G. E. , and R. H. Macarthur . 1959 A theoretical ecological model of size distributions among species of animals. Am. Nat. 93:117–125.

[ece31978-bib-0017] Kelt, D. A. , and M. D. Meyer . 2009 Body size frequency distributions in African mammals are bimodal at all spatial scales. Glob. Ecol. Biogeogr. 18:19–29.

[ece31978-bib-0018] Kim, S. H. , and S. V. Yi . 2007 Understanding relationship between sequence and functional evolution in yeast proteins. Genetica 131:151–156. 17160620 10.1007/s10709-006-9125-2

[ece31978-bib-0019] Knouft, J. H. 2004 Latitudinal variation in the shape of the species body size distribution: an analysis using freshwater fishes. Oecologia 139:408–417. 15069632 10.1007/s00442-004-1510-x

[ece31978-bib-0020] Kozlowski, J. , and A. T. Gawelczyk . 2002 Why are species' body size distributions usually skewed to the right? Funct. Ecol. 16:419–432.

[ece31978-bib-0021] Loder, N. , T. M. Blackburn , and K. J. Gaston . 1997 The slippery slope: towards an understanding of the body size frequency distribution. Oikos 78:195–201.

[ece31978-bib-0022] Marquet, P. A. , and H. Cofre . 1999 Large temporal and spatial scales in the structure of mammalian assemblages in South America: a macroecological approach. Oikos 85:299–309.

[ece31978-bib-0023] May, R. M. 1978 The dynamics and diversity of insect faunas Pp. 188–204 *in* Mound L. A. and Waloff N., eds. Diversity of insect faunas. Blackwell, Oxford.

[ece31978-bib-0024] May, R. M. 1986 The search for patterns in the balance of nature – advances and retreats. Ecology 67:1115–1126.

[ece31978-bib-0025] Morales‐Castilla, I. , M. A. Olalla‐Tarraga , A. Purvis , B. A. Hawkins , and M. A. Rodriguez . 2012 The imprint of cenozoic migrations and evolutionary history on the biogeographic gradient of body size in new world mammals. Am. Nat. 180:246–256. 22766934 10.1086/666608

[ece31978-bib-0026] Olson, V. A. , R. G. Davies , C. D. L. Orme , G. H. Thomas , S. Meiri , T. M. Blackburn , et al. 2009 Global biogeography and ecology of body size in birds. Ecol. Lett. 12:249–259. 19245587 10.1111/j.1461-0248.2009.01281.x

[ece31978-bib-0027] Peters, R. H. 1983 The Ecological Implications of Body Size. Cambridge Univ. Press, Cambridge, U.K.

[ece31978-bib-0028] Raia, P. , F. Carotenuto , and S. Meiri . 2010 One size does not fit all: no evidence for an optimal body size on islands. Glob. Ecol. Biogeogr. 19:475–484.

[ece31978-bib-0029] Rodriguez, M. A. , M. A. Olalla‐Tarraga , and B. A. Hawkins . 2008 Bergmann's rule and the geography of mammal body size in the Western Hemisphere. Glob. Ecol. Biogeogr. 17:274–283.

[ece31978-bib-0030] Smith, F. A. , and S. K. Lyons . 2011 How big should a mammal be? A macroecological look at mammalian body size over space and time. Philos. Trans. R. Soc. B Biol. Sci. 366:2364–2378. 10.1098/rstb.2011.0067 PMC3130437 21768152

[ece31978-bib-0031] Smith, F. A. , J. H. Brown , J. P. Haskell , S. K. Lyons , J. Alroy , E. L. Charnov , et al. 2004 Similarity of mammalian body size across the taxonomic hierarchy and across space and time. Am. Nat. 163:672–691. 15122486 10.1086/382898

[ece31978-bib-0032] Thibault, K. M. , E. P. White , A. H. Hurlbert , and S. K. M. Ernest . 2011 Multimodality in the individual size distributions of bird communities. Glob. Ecol. Biogeogr. 20:145–153.

[ece31978-bib-0033] Ulrich, W. , and C. Fiera . 2010 Environmental correlates of body size distributions of European springtails (Hexapoda: Collembola). Glob. Ecol. Biogeogr. 19:905–915.

[ece31978-bib-0034] White, E. P. , B. J. Enquist , and J. L. Green . 2008 On estimating the exponent of power‐law frequency distributions. Ecology 89:905–912. 18481513 10.1890/07-1288.1

[ece31978-bib-0035] Zar, J. H. 1999 Biostatistical analysis, 4th ed. Prentice Hall, Upper Saddle River

