# Supplementary material for: The role of local versus biogeographical processes in influencing diversity and body‐size variation in mammal assemblages
Source: Ecol Evol. 2016 Feb 3;6(5):1447–56. doi: 10.1002/ece3.1978 (PMC4775520; doi:10.1002/ece3.1978)
Supplement: Supplementary file 1 — Appendix S1 List of localities used in the analysis. [file ECE3-6-1447-s001.docx]

*Ecology and Evolution*

SUPPORTING INFORMATION

**The role of local vs. biogeographical processes in influencing diversity and body size variation in mammal assemblages**

Luiz Carlos S. Lopez, Marcos S. L. Figueiredo, Maria Paula de Aguiar Fracasso, Daniel Oliveira Mesquita, Ulisses Umbelino Anjos and Carlos Eduardo Viveiros Grelle

Appendix S1: List of localities used in the analysis

| Locality | Country | Continent | LatDec | LongDec | Source * |
| --- | --- | --- | --- | --- | --- |
| Niokolo-Koba NP | Senegal | AFR | 13.0667 | -12.7167 | BIWPA |
| Kainji Lake | Nigeria | AFR | 10.3667 | 4.5500 | Kelt & Meyer 2009 |
| Yankari Lake | Nigeria | AFR | 9.7544 | 10.5103 | Kelt & Meyer 2009 |
| Bale Mountains NP | Ethiopia | AFR | 6.8842 | 39.7331 | Kelt & Meyer 2009 |
| Harenna Forest | Ethiopia | AFR | 6.5212 | 39.2341 | Kelt & Meyer 2009 |
| Nechisar NP | Ethiopia | AFR | 6.0333 | 37.5833 | BIWPA |
| Taï NP | Ivory Coast | AFR | 5.7500 | -7.1167 | BIWPA |
| Dja Faunal Reserve | Cameroon | AFR | 3.0000 | 13.0000 | BIWPA |
| Noubale-Ndoki NP | Congo R | AFR | 2.4667 | 16.4500 | Kelt & Meyer 2009 |
| Odzala NP | Congo R | AFR | 0.8000 | 14.9333 | Kelt & Meyer 2009 |
| Makokou | Gabon | AFR | 0.5667 | 12.8667 | Kelt & Meyer 2009 |
| Kibale Forest NP | Uganda | AFR | 0.5000 | 30.4000 | Kelt & Meyer 2009 |
| Mount Kenya NP | Kenya | AFR | -0.1239 | 37.3367 | BIWPA |
| Kora NP | Kenya | AFR | -0.2086 | 38.7361 | BIWPA |
| Kahuzi-Biéga NP | Congo DR | AFR | -2.5000 | 28.7500 | BIWPA |
| Amboseli NP | Kenya | AFR | -2.6414 | 37.2481 | BIWPA |
| Lake Manyara NP | Tanzania | AFR | -3.5000 | 35.8333 | BIWPA |
| Mahale Mountains NP | Tanzania | AFR | -6.2667 | 29.9333 | BIWPA |
| Nkasa Rupara NP | Namibia | AFR | -18.3833 | 23.6167 | BIWPA |
| Wankie NP | Zimbabwe | AFR | -18.7350 | 26.9550 | Kelt & Meyer 2009 |
| Etosha NP | Namibia | AFR | -18.9453 | 15.8978 | BIWPA |
| Namib-Naukluft Park | Namibia | AFR | -24.5464 | 15.3297 | BIWPA |
| Malolotja NR | Swaziland | AFR | -26.0980 | 31.0990 | BIWPA |
| Mlawula NR | Swaziland | AFR | -26.1833 | 31.9833 | BIWPA |
| Naute Dam | Namibia | AFR | -26.9313 | 17.9383 | BIWPA |
| Qwa Qwa NP | South Africa | AFR | -28.4281 | 28.6940 | Kelt & Meyer 2009 |
| Golden Gate Highlands NP | South Africa | AFR | -28.5244 | 28.6208 | Kelt & Meyer 2009 |
| Augrabies Falls NP | South Africa | AFR | -28.5911 | 20.3383 | Kelt & Meyer 2009 |
| Hester Malan Reserve | South Africa | AFR | -29.6667 | 18.0333 | Kelt & Meyer 2009 |
| West Coast NP | South Africa | AFR | -33.1208 | 18.0667 | Kelt & Meyer 2009 |
| Arctic National Wildlife Refuge | USA | NAM | 68.7500 | -143.5000 | BIWPA |
| Vuntut NP | Canada | NAM | 68.3069 | -140.0475 | BIWPA |
| Nahanni NP | Canada | NAM | 61.0833 | -123.6000 | BIWPA |
| Wrangell–St. Elias NP | USA | NAM | 61.0000 | -142.0000 | BIWPA |
| Banff NP | Canada | NAM | 51.1667 | -115.5500 | BIWPA |
| Grasslands NP | Canada | NAM | 49.1769 | -107.4258 | BIWPA |
| Cook Co., MN | USA | NAM | 47.9167 | -90.5500 | Brown & Nicoletto 1991 |
| Lake Superior Provincial Park | Canada | NAM | 47.5967 | -84.7414 | BIWPA |
| Norman Co., MN | USA | NAM | 47.3300 | -96.4600 | Brown & Nicoletto 1991 |
| Kouchibouguac NP | Canada | NAM | 46.8497 | -64.9669 | BIWPA |
| La Mauricie NP | Canada | NAM | 46.8000 | -72.9667 | BIWPA |
| Washington Co., MN | USA | NAM | 45.0400 | -92.8900 | Brown & Nicoletto 1991 |
| Lac Qui Parle Co., MN | USA | NAM | 45.0000 | -96.1800 | Brown & Nicoletto 1991 |
| Yellowstone NP | USA | NAM | 44.6000 | -110.5000 | BIWPA |
| Green Mountains, VT | USA | NAM | 43.9500 | -73.0667 | Brown & Nicoletto 1991 |
| DeSoto National Wildlife Refuge | USA | NAM | 41.5237 | -96.0179 | BIWPA |
| Ligonier Valley, PA | USA | NAM | 40.3100 | -79.4700 | Brown & Nicoletto 1991 |
| Sagehen, CA | USA | NAM | 39.4325 | -120.2369 | Brown & Nicoletto 1991 |
| Konza Prairie, KS | USA | NAM | 39.0931 | -96.5586 | Brown & Nicoletto 1991 |
| Zion NP | USA | NAM | 37.2026 | -112.9879 | BIWPA |
| Lake Meredith NP | USA | NAM | 35.7147 | -101.5528 | BIWPA |
| Bernalillo Co., NM | USA | NAM | 35.3094 | -106.5519 | Brown & Nicoletto 1991 |
| Wheeler National Wildlife Refuge | USA | NAM | 34.5577 | -86.9516 | BIWPA |
| Deep Canyon, CA | USA | NAM | 33.5833 | -116.3333 | Brown & Nicoletto 1991 |
| Cape Romain NWR | USA | NAM | 32.9925 | -79.5639 | BIWPA |
| White Sands, NM | USA | NAM | 32.3842 | -106.4978 | Brown & Nicoletto 1991 |
| Cochise Co., AZ | USA | NAM | 32.1136 | -109.9217 | Brown & Nicoletto 1991 |
| Alto Golfo de California | Mexico | NAM | 31.7400 | -114.6600 | BIWPA |
| Animas Mountain, NM | USA | NAM | 31.5695 | -108.7887 | Brown & Nicoletto 1991 |
| Big Thicket BR | USA | NAM | 30.5467 | -94.3403 | BIWPA |
| Mapimí BR | Mexico | NAM | 26.6914 | -103.7456 | BIWPA |
| Big Cypress NP | USA | NAM | 25.8589 | -81.0339 | BIWPA |
| Chamela BS | Mexico | NAM | 19.5300 | -105.0733 | Brown & Nicoletto 1991 |
| Montes Azules | Mexico | NAM | 16.5000 | -91.0000 | Medellín 1994 |
| La Selva | Costa Rica | NAM | 10.4333 | -83.8833 | Voss & Emmons 1996 |
| Chiquibul NP | Belize | SAM | 16.7103 | -88.9375 | BIWPA |
| La Amistad | Panama / Costa Rica | SAM | 9.4071 | -82.9388 | BIWPA |
| Kartabo | Guyana | SAM | 6.3833 | -58.6833 | Voss & Emmons 1996 |
| Iwokrama Forest | Guyana | SAM | 4.5000 | -59.0000 | BIWPA |
| Baixo Arataye | French Guyana | SAM | 4.0833 | -52.6667 | Voss & Emmons 1996 |
| Rio Cunucunuma | Venezuela | SAM | 3.6500 | -65.7667 | Voss & Emmons 1996 |
| Yasuni BR | Ecuador | SAM | -1.0833 | -75.9167 | BIWPA |
| MCSE | Brazil | SAM | -2.5000 | -60.0000 | Voss & Emmons 1996 |
| Rio Xingu | Brazil | SAM | -3.6500 | -52.3667 | Voss & Emmons 1996 |
| alto Marañon | Peru | SAM | -4.4333 | -78.2833 | Patton et al. 1982 |
| Urucu | Brazil | SAM | -4.8333 | -65.2667 | Peres 1999 |
| PEMVS | Brazil | SAM | -8.3668 | -36.0270 | Sousa et al. 2004 |
| Balta | Peru | SAM | -10.1333 | -71.2167 | Voss & Emmons 1996 |
| Cocha Cashu e Pakitza | Peru | SAM | -11.9000 | -71.3667 | Voss & Emmons 1996 |
| Cuzco amazonico | Peru | SAM | -12.5500 | -69.0500 | Voss & Emmons 1996 |
| Noel Kempff Mercado NP | Bolivia | SAM | -14.2667 | -60.8667 | BIWPA |
| Águas Emendadas ES | Brazil | SAM | -15.5591 | -47.6063 | BIWPA |
| IBGE | Brazil | SAM | -15.9452 | -47.8357 | Fonseca & Redford, 1985 |
| Lauca NP | Chile | SAM | -18.2444 | -69.3539 | BIWPA |
| Serra dos Órgãos NP | Brazil | SAM | -22.4454 | -42.9998 | Ollifiers et al. 2007; Cunha 2007 |
| Río Pilcomayo NP | Argentina | SAM | -25.0667 | -58.1167 | BIWPA |
| Iguazú NP | Argentina | SAM | -25.6167 | -54.3333 | BIWPA |
| Bañados del Este | Uruguay | SAM | -33.9333 | -53.5000 | BIWPA |
| Otamendi NP | Argentina | SAM | -34.2342 | -58.8861 | BIWPA |
| Lanín NP | Argentina | SAM | -40.1603 | -71.3575 | BIWPA |
| Los Alerces NP | Argentina | SAM | -42.8075 | -71.8989 | BIWPA |
| Bosques Petrificados MN | Argentina | SAM | -47.6666 | -68.1667 | BIWPA |
| Torres del Paine NP | Chile | SAM | -50.9831 | -72.9664 | BIWPA |

*BIWPA= http://www.ice.ucdavis.edu/bioinventory/bioinventory.html
